# Whole-Genome Sequence Analysis of *Bombella intestini *LMG 28161^T^, a Novel Acetic Acid Bacterium Isolated from the Crop of a Red-Tailed Bumble Bee, *Bombus lapidarius*

**DOI:** 10.1371/journal.pone.0165611

**Published:** 2016-11-16

**Authors:** Leilei Li, Koen Illeghems, Simon Van Kerrebroeck, Wim Borremans, Ilse Cleenwerck, Guy Smagghe, Luc De Vuyst, Peter Vandamme

**Affiliations:** 1 Laboratory of Microbiology, Faculty of Sciences, Ghent University, K. L. Ledeganckstraat 35, B-9000 Ghent, Belgium; 2 Research Group of Industrial Microbiology and Food Biotechnology, Faculty of Sciences and Bioengineering Sciences, Vrije Universiteit Brussel, Pleinlaan 2, B-1050 Brussels, Belgium; 3 BCCM/LMG Bacteria Collection, Faculty of Sciences, Ghent University, K. L. Ledeganckstraat 35, B-9000 Ghent, Belgium; 4 Laboratory of Agrozoology, Department of Crop Protection, Faculty of Bioscience Engineering, Ghent University, Coupure links 653, B-9000 Ghent, Belgium; University of Milan, ITALY

## Abstract

The whole-genome sequence of *Bombella intestini* LMG 28161^T^, an endosymbiotic acetic acid bacterium (AAB) occurring in bumble bees, was determined to investigate the molecular mechanisms underlying its metabolic capabilities. The draft genome sequence of *B*. *intestini* LMG 28161^T^ was 2.02 Mb. Metabolic carbohydrate pathways were in agreement with the metabolite analyses of fermentation experiments and revealed its oxidative capacity towards sucrose, D-glucose, D-fructose and D-mannitol, but not ethanol and glycerol. The results of the fermentation experiments also demonstrated that the lack of effective aeration in small-scale carbohydrate consumption experiments may be responsible for the lack of reproducibility of such results in taxonomic studies of AAB. Finally, compared to the genome sequences of its nearest phylogenetic neighbor and of three other insect associated AAB strains, the *B*. *intestini* LMG 28161^T^ genome lost 69 orthologs and included 89 unique genes. Although many of the latter were hypothetical they also included several type IV secretion system proteins, amino acid transporter/permeases and membrane proteins which might play a role in the interaction with the bumble bee host.

## Background

Acetic acid bacteria (AAB) are best known for their production of acetic acid from ethanol during vinegar and cocoa bean fermentation [1–3]. Some AAB are also of interest to the industry because of their capacity to produce cellulose or other chemicals, such as L-sorbose involved in the synthesis of vitamin C [4]. Furthermore, AAB occur as plant growth-promoting bacteria [5], insect endosymbionts [6], and as spoilers of many kinds of beverages such as wine and beer [7]. AAB are classified in the family *Acetobacteraceae* within the *Alphaproteobacteria*. Recent studies of the symbiotic relationship between AAB and several insect hosts have revealed that this symbiosis relies on sugar-based diets such as nectar, fruit sugar, or phloem sap [6, 8]. During a study of the bumble bee and honey bee gut microbiota, an *Acetobacteraceae* operational taxonomic unit, referred to as Alpha-2.2, was repeatedly detected in the digestive tract of honey bees (*Apis* spp.) and bumble bees (*Bombus* spp.) [9–13]. These bacteria were categorized as one of the core bacteria in *Bombus bimaculatus* [9] and its presence in wild bumble bees (*Bombus*) was positively associated with *Crithidia* infection [9]. The Alpha-2.2 strain LMG 28161^T^ was recently isolated from the crop of a red-tailed bumble bee, *Bombus lapidarius;* it showed 97–99% pairwise 16S rRNA gene sequence identity to Alpha-2.2 sequences and was formally classified into a novel genus as *Bombella intestini* [14]. Another Alpha-2.2 isolate, A29, was recently described as “*Parasaccharibacter apium*” and showed 98.9% 16S rRNA sequence similarity with *B*. *intestini* LMG 28161^T^; it was proven to be helpful in improving honey bee resistance to *Nosema* infections [13]. *B*. *intestini* LMG 28161^T^ shows distinctive phenotypic features from other AAB, such as *Gluconobacter* and *Acetobacter* [14]. In the present study, the genomic characteristics of *B*. *intestini* LMG 28161^T^ were examined through a whole-genome sequencing approach and its capability to oxidize the main components of nectar and honey, *i*.*e*. sucrose, glucose and fructose [6] and D-mannitol, a six-carbon sugar alcohol that is widely distributed in plants, were explored [15] through cultivation experiments under both aerobic and micro-aerobic conditions. Genomes of three insect associated AAB strains, *i*. *e*. *Asaia platycodi* SF2.1, *Commensalibacter intestini* A911, *Saccharibacter* sp. AM169 and of its nearest phylogenetic neighbor, *Saccharibacter floricola* DSM 15669^T^ (an organism isolated from the pollen of Japanese flowers) were used in an ortholog analysis to explore the genetic symbiotic traits in *B*. *intestini*.

## Results and Discussion

### General genome features

The genome sequencing of *B*. *intestini* strain LMG 28161^T^, an endosymbiotic acetic acid bacterium occurring in bumble bees, yielded more than 6 million reads of 2 x 100 bp with a genome coverage of 299.0 x. All reads were assembled into 12 contigs of 1,402 to 670,914 nucleotides. Automated gene prediction and annotation of the assembled genome sequences resulted after manual curation in a draft genome of 2.02 Mb with an average G + C content of 54.9%. The latter value was identical to the DNA G+C content determined through an enzymatic degradation method [16] and separation of the nucleoside mixture through high-performance liquid chromatography [14]. The general genome features are summarized in Table 1. The genome size of *B*. *intestini* is most similar to that of *Saccharibacter* sp. AM169 (1.9 Mb) which was isolated from the honey bee *Apis mellifera* [17]; however, it is smaller than that of most other AAB that have been determined (2.7–3.9 Mb) [2, 18–20]. The reduced genome size may be indicative for gene loss, which can cause reduced functional capabilities, a typical feature of niche-specific microorganisms, such as bacterial endosymbionts and may suggest that *B*. *intestini* has adapted to the bumble bee digestive tract [13, 21–22].

**Table 1:** General genome features of *Bombella intestini* LMG 28161^T^.

Organism	*Bombella intestini* LMG 28161^T^
Genome size (bp)	2,023,177
Number of contigs	12
G+C%	54.9
CDS	1,574
tRNAs	50
rRNAs	3
Accession number	PRJNA235371

No plasmids were found during the assembly. The final annotation resulted in 1574 coding sequences (CDSs) and 54 RNA sequences, including three rRNA genes (5S, 16S and 23S), 50 tRNA genes, and one non-coding RNA. Three clustered regularly interspaced short palindromic repeats (CRISPRs) were found on contig 1, contig 2, and contig 5. CRISPR-associated CDSs were found on contig 1 (AL01_08840, AL01_08855) and contig 5 (AL01_03255, AL01_03260, AL01_03265). The three CRISPR arrays are 233 bp, 1736 bp and 1736 bp in length, with 3, 28 and 28 spacers, respectively. The draft genome was submitted to NCBI under the BioProject PRJNA235371.

### Metabolic pathways of carbohydrates

**Fig 1:**
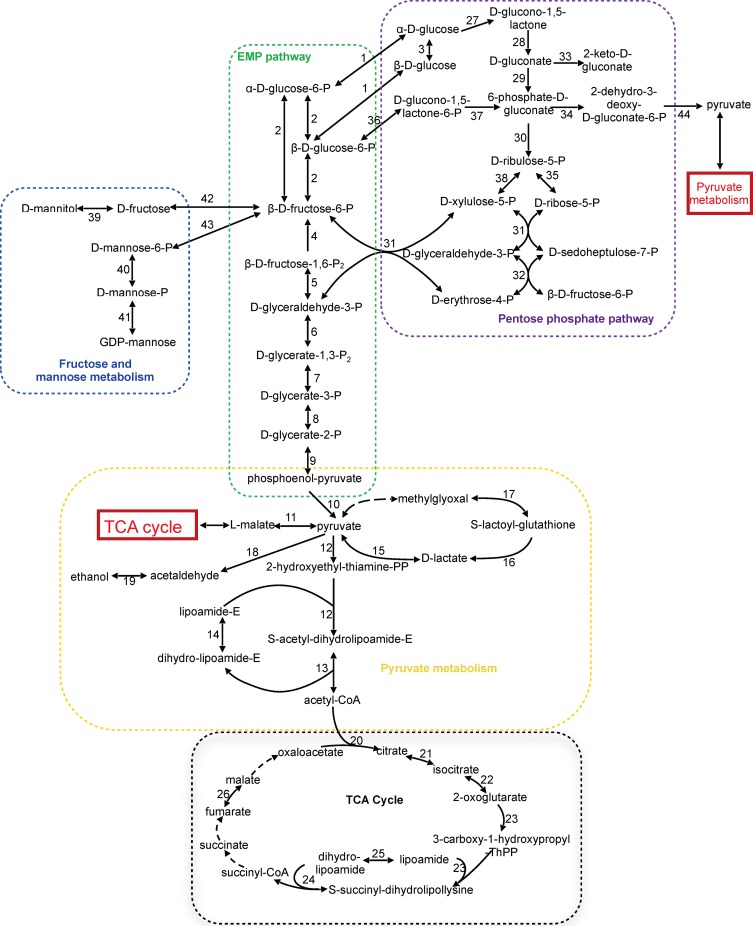
Central metabolic pathways of *Bombella intestini* LMG 28161^T^. Dashed arrows represent missing steps in the pathways due to the absence of corresponding genes. Genes encoding enzymes that catalyze the reaction between pyruvate and methylglyoxal in pyruvate metabolism and those that catalyze the conversion of succinyl-CoA to succinate, succinate to fumarate and malate to oxaloacetate in the TCA cycle were not found in the draft genome. 1, glucokinase (AL01_01675); 2, glucose-6-phosphate isomerase (AL01_06115); 3, aldose epimerase (AL01_04275); 4, fructose 1, 6-bisphosphatase (AL01_08695); 5, fructose-bisphosphate aldolase (AL01_06890); 6, glyceraldehyde-3-phosphate dehydrogenase (AL01_03750); 7, phosphoglycerate kinase (AL01_03755); 8, phosphoglycerate mutase (AL01_07435, AL01_07655); 9, enolase (AL01_00860); 10, pyruvate kinase (AL01_00625); 11, malate dehydrogenase (AL01_05845); 12, pyruvate dehydrogenase (AL01_00915, AL01_00920, AL01_03860); 13, pyruvate dehydrogenase E2 (AL01_00925); 14, dihydrolipoamide dehydrogenase (AL01_00930); 15, lactate dehydrogenase (AL01_06935); 16, hydroxyacylglutathione hydrolase (AL01_04950); 17, lactoylgluthathione lyase (AL01_00090); 18, pyruvate decarboxylase (AL01_08375); 19, alcohol dehydrogenase (AL01_01980, AL01_07015); 20, citrate synthase (AL01_06255); 21, aconitate hydratase 1 (AL01_06260); 22, NADP+-dependent isocitrate dehydrogenase (AL01_06250); 23, 2-oxoglutarate dehydrogenase E1 (AL01_08340); 24, dihydrolipoyllysine succinyltransferase (AL01_07740); 25, dihydrolipoamide dehydrogenase (AL01_00930); 26, fumarate hydratase (AL01_05840); 27, PQQ-dependent glucose dehydrogenase (AL01_09305); 28, gluconolactonase (AL01_06230); 29, lactate dehydrogenase (AL01_06935); 30, phosphogluconate dehydrogenase (AL01_06120); 31, transketolase (AL01_06110); 32, transaldolase (AL01_06115); 33, gluconate 2-dehydrogenase (AL01_07015); 34, 6-phosphogluconate dehydrogenase (AL01_06120); 35, ribose-5-phosphate isomerase (AL01_06135); 36, glucose-6-phosphate dehydrogenase (AL01_02790); 37, 6-phosphogluconolactonase (AL01_06130); 38, ribulose-phosphate 3-epimerase (AL01_09060); 39, polyol:NADP oxidoreductase (AL01_07080); 40, phosphomannomutase (AL_0102400); 41, mannose-1-phosphate guanyltransferase (AL01_07360); 42, carbohydrate kinase (AL01_03675); 43, mannose06-phosphate isomerase (AL01_00140); 44, 2-dehydro-3-deoxyphosphogluconate aldolase (AL01_04330).

Based on the annotated draft genome, central metabolic pathways including the Embden-Meyerhof-Parnas (EMP) pathway, the pentose-phosphate pathway (PPP), the pyruvate pathway, and the tricarboxylic acid (TCA) cycle were reconstructed (Fig 1).

All genes encoding the enzymes of the EMP pathway were identified, except for the phosphofructokinase-coding gene, suggesting incomplete glycolysis. The absence of this gene in AAB has been reported before for *Gluconobacter oxydans* 621H, *Acetobacter pasteurianus* IFO 3283, and *Gluconacetobacter diazotrophicus* Pal5^T^ [2, 18–20].

All genes encoding the enzymes of the PPP were identified, enabling degradation of hexoses such as glucose and fructose via this pathway. Uptake of hexoses appeared possible through a sugar transporter (AL01_05795 and AL01_06590) or a D-galactose transporter encoded by *galP* (AL01_03445, AL01_03450 and AL01_02185), which both belong to the major facilitator superfamily (MFS) [19, 23]. Phenotypic tests, using a method described previously [24], revealed that *B*. *intestini* LMG 28161^T^ was capable to produce acid from several carbohydrates, including sucrose, D-glucose, D-fructose, D-galactose, D-mannitol, and D-mannose [14]. A polyol oxidoreductase (AL01_07080) enabling the conversion of D-mannitol into D-fructose was also found, as well as genes encoding enzymes that catalyze D-mannose utilization (Fig 1). This supported the previous observation that *B*. *intestini* LMG LMG 28161^T^ is able to produce acid from D-mannitol and D-mannose [14].

**Fig 2:**
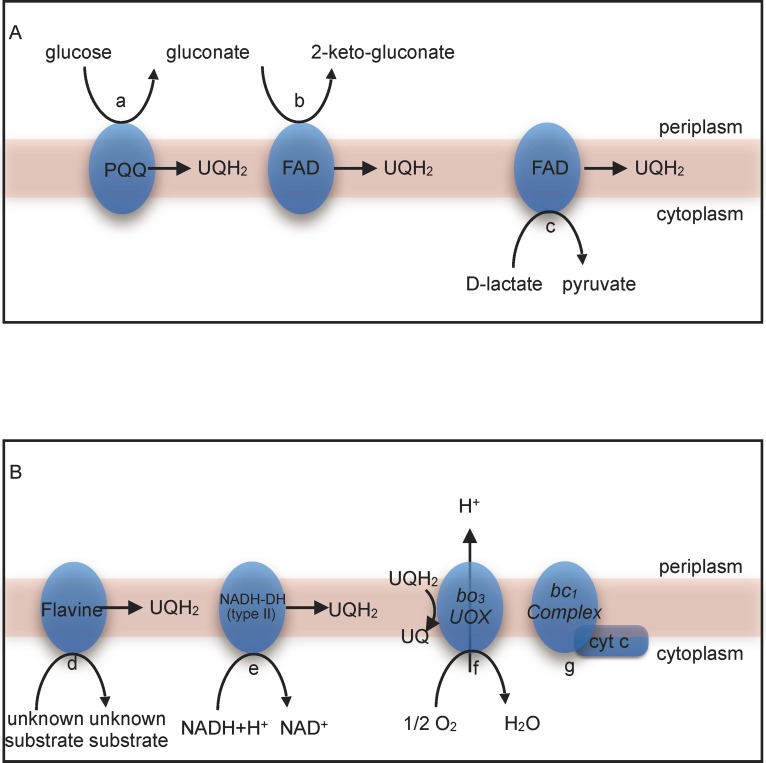
**A) Membrane bound dehydrogenase and B) respiratory chain of *Bombella intestini* LMG 28161**^**T**^. a, membrane-bound glucose dehydrogenase (AL01_09305); b, gluconate 2-dehydrogenase (AL01_07015); c, membrane-bound lactate dehydrogenase (AL01_06935); d, electron transfer flavoprotein-ubiquinone oxidoreductase (AL01_08300); e, type II NADH dehydrogenase (AL01_05990); f, cytochrome bo3 ubiquinol oxidase (AL01_00470, AL01_00475, AL01_00480 and AL01_00485); g, ubiquinol-cytochrome c reductase (bc1 complex) (AL01_05885, AL01_08145, AL01_08150); h, Cyt C, cytochrome c (AL01_05875).

D-gluconate could be oxidized into 2-keto-D-gluconate by a membrane-bound gluconate 2-dehydrogenase (AL01_07015) (Fig 2A). A gene encoding gluconate-5-dehydrogenase was not found. This was in accordance with the previous observation that this microorganism can produce 2-keto-D-gluconate but not 5-keto-D-gluconate [14]. A general alcohol dehydrogenase-coding gene (AL01_01980) was found, but no gene encoding an enzyme for the oxidation of acetaldehyde into acetate. This supported the phenotypic inability of this strain to produce acetate from ethanol [14]. Furthermore, a gene encoding glycerol kinase was not detected, suggesting that glycerol could not be transferred into the cell and further utilized. This explained why this microorganism can not grow on or produce acid from glycerol [14].

*B*. *intestini* LMG 28161^T^ appeared to possess an incomplete TCA cycle. Genes coding for enzymes converting succinyl-CoA into succinate, succinate into fumarate, and malate into oxaloacetate were not identified. However, L-asparagine permease (AL01_09015) and L-aspartate oxidase (AL01_04960) were identified. The former could enable the microorganism to take up L-asparagine from the environment, which could then be hydrolyzed to L-aspartate. L-aspartate oxidase is a flavoprotein that acts on the CH-NH_2_ group of donors with oxygen as electron acceptor [4]. Oxygen can be replaced by fumarate as electron acceptor, yielding succinate [23]. The ability of the enzyme to use both oxygen and fumarate in cofactor re-oxidation enables it to function under both aerobic and anaerobic conditions [23]. L-aspartate could be converted by aspartate aminotransferase (AL01_03035) into oxaloacetate to join the TCA cycle. As for fumarate, it could also be derived from L-aspartate via two different two-step reactions with adenylosuccinate or L-argininosuccinate as intermediates, catalyzed by adenylosuccinate synthetase (AL01_06765), adenylosuccinate lyase (AL01_00960), argininosuccinate synthase (AL01_09265) and argininosuccinate lyase (AL01_02240). Although the three above-mentioned substrates of the TCA cycle could be generated by other reactions, the amount of energy generated through the TCA cycle may be rather limited, as in a complete TCA cycle the three enzymatic reactions catalyzed by these three missing enzymes are accompanied by the generation of GTP, FADH_2_ or NADH [4].

### Membrane-bound dehydrogenases and respiratory chain

Compared to other genome-sequenced AAB strains, *B*. *intestini* LMG 28161^T^ did not possess many membrane-bound dehydrogenases, as only three were found (Fig 2A), namely a cofactor pyrroloquinoline quinone (PQQ)-dependent glucose dehydrogenase (AL01_09305) allowing the conversion of glucose into gluconate, a flavine adenine dinucleotide (FAD)-dependent gluconate 2-dehydrogenase (AL01_07015) allowing the conversion of gluconate into 2-keto-gluconate, and a FAD-dependent D-lactate oxidase (AL01_06935) allowing the conversion of D-lactate into pyruvate. A *pqqBCDE* operon (AL01_07315, AL01_07320, AL01_07325 and AL01_07330) encoding proteins for the biosynthesis of the cofactor PQQ was found. In the genomes of *G*. *oxydans* 621H and *A*. *pasteurianus* 386B, a *pqqABCDE* operon responsible for PPQ biosynthesis is present [2]. It has been shown previously that a *pqqA* mutant of *G*. *oxydans* 621H is unable to grow on D-mannitol, D-glucose, or glycerol as the sole energy source [25]. *Bombella intestini* LMG 28161^T^ could grow on D-glucose and D-mannitol as the sole energy source (see below), which indicates that *pqqA* was not vital for this strain.

Genes encoding ubiquinol-cytochrome *c* reductase (*bc*_*1*_ complex) (AL01_05885, AL01_08145, AL01_08150) and cytochrome *c* (AL01_05875) were found in the genome, but not cytochrome *c* oxidase-encoding genes. Genes encoding a type II NADH dehydrogenase (AL01_05990) and a NAD(P)H:ubiquinone oxidoreductase (AL01_07780) were present in the genome. Both these enzymes catalyze electron transfer from NADH to ubiquinone [4]. A flavoprotein-ubiquinone oxidoreductase (AL01_08300) could catalyze electron transfer from flavoprotein to ubiquinone. The reduced product, ubiquinol, could diffuse within the membrane and be re-oxidized by cytochrome *bo*_*3*_ ubiquinole oxidase (AL01_00470, AL01_00475, AL01_00480 and AL01_00485) (Fig 2B). A previous study has suggested that AAB acquired ubiquinol oxidase from *β/γ-Proteobacteria* via horizontal gene transfer and created afterwards a truncated respiratory chain in which electron transfer to oxygen occurs via ubiquinol oxidase directly, accepting electrons from ubiquinol [26]. The truncated respiratory chain would generate less energy, but allow rapid oxidations, which would be beneficial for AAB [26]. Cytochrome *bo*_*3*_ oxidase has been detected in other AAB genomes and shows a high affinity for oxygen, possibly allowing their survival in environments with low oxygen availability, such as the insect gut [17].

### Amino acid metabolism and nitrogen metabolism

Pathways for all proteinogenic amino acids except alanine were present. L-alanine could not be converted from L-aspartate due to the absence of aspartate 4-decarboxylase and might be taken from the environment via an amino acid transporter or permease. Glutamate can be converted to ornithine and enter the urea cycle, where ornithine as well as aspartate are eventually converted to fumarate and enter the citrate cycle. Nitrogen fixation pathways were absent; ammonia can be incorporated into glycine by glycine synthase or to cyclic amidines by NAD synthase.

### Bumble bee endosymbionts-related features

An ortholog analysis of *B*. *intestini* LMG 28161^T^, *A*. *platycodi* SF2.1, *C*. *intestini* A911, *Saccharibacter* sp. AM169 and *S*. *floricola* DSM 15669^T^ carried out in OrthoMCL resulted in 1397 ortholog groups, including 894 core orthologs. In total 69 orthologs were shared among the four reference genomes but not present in *B*. *intestini* LMG 28161^T^, including cytochrome d ubiquinol oxidase subunit I, II, 39 functional genes and 28 hypothetical genes (S1 Table). Compared to the above mentioned reference strains, *B*. *intestini* LMG 28161^T^ possessed 86 unique genes, of which 63 were hypothetical protein coding sequences and 23 were functional genes (S2 Table). Among the 23 functional genes, genes encoding for five type IV secretion system proteins, three amino acid transporter/permeases and three membrane proteins were unique to *B*. *intestini*. ABC transporters and type IV secretion systems have been reported to be involved in the cross talk between endosymbionts and their insect hosts [17, 21, 27–28]. Multiple CDSs associated to ABC transporters were identified (Table 2). A signal recognition particle (SRP) complex (AL01_03075 and AL01_06750), which recognizes and targets specific proteins on the plasma membrane, was also present. The restriction modification system to degrade foreign DNA, which has been found in the genomes of two additional *Bombus* endosymbionts, *Gilliamella apicola* and *Snodgrasella alvi* [21], was not detected in the genome of *B*. *intestini* LMG 28161^T^; yet, CRISPR elements were present and may be used in the defense against bacteriophages.

**Table 2:** CDSs related to ABC transporters found in the draft genome of *B*. *intestini* LMG 28161^T^.

Gene product	Locus tag
ABC transporter	AL01_07240, AL01_02455
ABC transporter permease	AL01_07235, AL01_07895, AL01_01955, AL01_02560, AL01_04765, AL01_08515
ABC transporter substrate-binding protein	AL01_03865
ABC transporter ATP-binding protein	AL01_06795, AL01_00180, AL01_00230, AL01_01625, AL01_01630, AL01_01950
Multidrug ABC transporter	AL01_08210, AL01_08950
Multidrug ABC transporter substrate-binding protein	AL01_02460
Multidrug ABC transporter ATPase	AL01_05575
Peptide ABC transporter permease	AL01_07685
Amino acid ABC transporter permease	AL01_05630
Branched-chain amino acid ABC transporter permease	AL01_05150
Iron-siderophore ABC transporter permease	AL01_00405
Iron ABC transporter substrate-binding protein	AL01_00400, AL01_01945
Iron ABC transporter ATP-binding protein	AL01_00410
Polyamine ABC transporter ATP-binding protein	AL01_07890
Glutamine ABC transporter ATP-binding protein	AL01_05625
Nitrate ABC transporter ATP-binding protein	AL01_01705
Organic solvent ABC transporter substrate-binding protein	AL01_02300
Phosphate ABC transporter substrate-binding protein	AL01_07215

### Metabolite analysis

**Fig 3:**
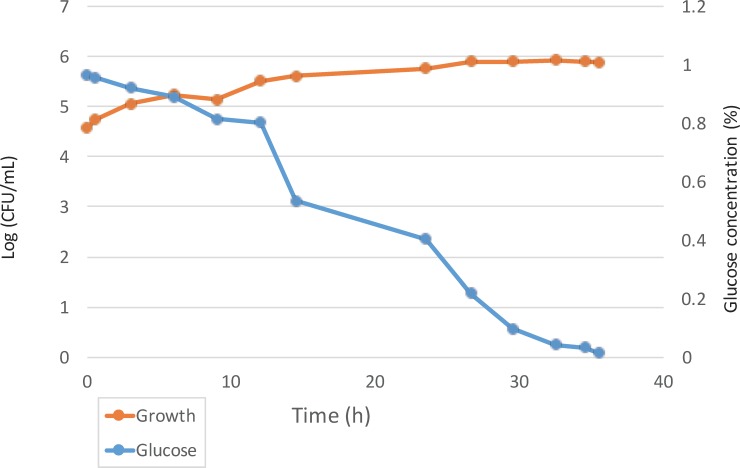
Growth of and D-glucose consumption by *Bombella intestini* LMG 28161^T^ during fermentation in basal medium with 1% (w/v) D-glucose. Glucose was consumed rapidly after 12h and was completely used after 36h. Growth of cells stabilized after some 27h.

During a monoculture fermentation experiment at 10-L scale, *B*. *intestini* LMG 28161^T^ oxidized D-glucose from the beginning of the fermentation, with a rapid drop after 12 h until D-glucose was completely depleted after 36 h (Fig 3). The biomass formation during fermentation was limited [from 4.5 to 5.9 log (CFU/mL)], which was probably due to the nutrient-limited composition of the medium used. The pH value of the medium decreased from 7.0 to 3.5 during fermentation, which was in accordance with the acid production from D-glucose [14].

Bumble bees feed on nectar, which comprises sucrose, fructose, glucose, and in some plants also D-mannitol [29] as the main components; glucose and mannitol are also often used as main carbon source in AAB growth media [1, 14]. Therefore, these carbohydrates may serve as carbon source for *B*. *intestini* during its endosymbiotic lifestyle. As the digestive tract of bees is a micro-aerobic environment, growth on sucrose, D-glucose, D-fructose, and D-mannitol was checked under both aerobic and micro-aerobic conditions in 50-mL glass bottle experiments. *Bombella intestini* LMG 28161^T^ was capable to utilize these four carbohydrates; under both aerobic and micro-aerobic conditions, D-glucose was consumed the most, followed by sucrose, however, sucrose and D-glucose were more consumed under aerobic than under micro-aerobic conditions, whereas D-fructose was utilized more under micro-aerobic conditions; for mannitol, no difference was seen (Table 3). In addition, 2-keto-D-gluconic acid was produced under both incubation conditions when sucrose and D-glucose were used as the sole carbon source. Gluconic acid and acetic acid were not found. The cultivation experiments therefore confirmed that *B*. *intestini* LMG 28161^T^ was able to produce 2-keto-D-gluconic acid through oxidation of glucose (and sucrose) under both aerobic and micro-aerobic conditions.

**Table 3:** Means and SD, comparison of means of carbohydrate consumption (T-test) by *Bombella intestini* LMG 28161^T^ under aerobic and micro-aerobic conditions. Means are tested for difference by Least Significant Difference (LSD) test. Means indicated by the same letter in a column do not differ (P = 0.05) according to LSD test.

Carbohydrates	Percentage amount of carbohydrates consumed under different cultivation conditions (%)		T-test
	Aerobic	Micro-aerobic	
Sucrose	34.95±1.811 ^a^	29.41±2.036 ^a^	0.0125*
Glucose	52.45±0.360 ^b^	46.32±1.190 ^b^	0.0039**
Fructose	15.02±5.147 ^c^	25.94±1.696 ^c^	0.0275*
D-mannitol	18.30±3.928 ^c^	20.40±1.681 ^d^	0.2313

* Significant at α = 0.05.

**Significant at α = 0.01.

During the 10-L fermentation experiment (aerobic conditions), D-glucose was depleted completely after 36 h of incubation, whereas during the 50-mL glass bottle experiments (aerobic conditions) none of the four carbohydrates investigated was depleted. In the latter case, growth mainly occurred at the surface. The difference in aeration might explain the difference in carbohydrate depletion during the two test systems. The lack of effective aeration in small-scale carbohydrate consumption experiments may explain the lack of reproducibility of such results often reported in taxonomic studies of AAB [14, 30, 31].

## Conclusion

*B*. *intestini* LMG 28161^T^, an endosymbiotic acetic acid bacterium occurring in bumble bees, carries a small genome of 2.02 Mb. The reconstructed metabolic pathways were congruent with the results of the fermentation experiments and with phenotypic features determined previously [14]. Compared to four other AAB genomes analyzed, the genome of *B*. *intestini* lost 69 orthologs that were shared among the other 4 strains. Simultaneously it included 89 unique genes, many of which were hypothetical. The unique genes in *B*. *intestini* included genes encoding for several type IV secretion system proteins, amino acid transporter/permeases and membrane proteins, which might play a role in the interaction with the bumble bee host. *B*. *intestini* LMG 28161^T^ was capable to oxidize sucrose, D-glucose, D-fructose, and D-mannitol, which are all present in nectar or honey, while it was incapable of oxidizing ethanol or glycerol, which are not available in the bumble bee gut. In addition, *B*. *intestini* showed a different preference in carbohydrates consumption under aerobic and micro-aerobic conditions, where sucrose and D-glucose were more preferred under aerobic condition, while D-fructose was utilized more under micro-aerobic conditions.

## Materials and Methods

### Strain cultivation, DNA extraction, genome sequencing, assembly, and annotation

*Bombella intestini* strain LMG 28161^T^ was cultivated in LMG medium 404 [5%, w/v, D-glucose; 1%, w/v, yeast extract (Oxoid) and 1.5%, w/v, agar] for DNA extraction at large scale, using the method of Wilson as modified previously [32]. The integrity of the DNA was evaluated on a 1.0% (w/v) agarose gel and the purity was checked by spectrophotometric measurements at 234, 260 and 280 nm. The DNA concentration was estimated with a Quantus™ fluorometer using a QuantiFluor®ONE ds DNA system kit (Promega Corporation, Madison, WI, USA). Library preparation and genome sequencing were performed by BaseClear BV (Leiden, The Netherlands). Paired-end sequence reads were generated using the Illumina HiSeq2500 system (Illumina Inc., San Diego, CA, USA). The initial *de novo* assembly of the raw reads into contigs was performed using the CLCgenomic workbench v6.5.1 (CLC Inc, Aarhus, Denmark).

Automated gene prediction and annotation of the assembled genome sequences were performed with GenDB v2.2 [33], the Rapid Annotations using Subsystems Technology (RAST) server [34], and the NCBI Prokaryotic Genomes Automatic Annotation Pipeline (PGAAP; http://www.ncbi.nlm.nih.gov/genomes/static/Pipeline.html). The PGAAP gene predictions and annotations were used as basis for the final annotation. They were manually curated for the coding sequences (CDSs) of interest using BLASTp (http://blast.ncbi.nlm.nih.gov/blast) and UniProt (http://www.uniprot.org), taking also into account the information from RAST and GenDB. Metabolic pathways were manually reconstructed using the information from the final annotation. The KEGG database [35] aided in the reconstruction of the pathways. CRISPRs were searched for using CRISPR Finder [36] and considered if they were classified as ‘confirmed’.

### Ortholog analysis

Four genomes of AAB were selected for the ortholog analysis, including three insect associated AAB strains, *Asaia platycodi* SF2.1, *Commensalibacter intestini* A911, *Saccharibacter* sp. AM169 and the nearest phylogenetic neighbor of *B*. *intestini*, *Saccharibacter floricola* DSM 15669^T^ (an organism isolated from the pollen of Japanese flowers) (Table 4). The analysis was carried out using OrthoMCL [37].

**Table 4:** List of bacterial genomes used for the ortholog studies.

Organism	Accession number	Origin
*Bombella intestini* LMG 28161^T^	PRJNA235371	Crop of a bumble bee
*Asaia platycodi* SF2.1	CBLX010000001:27	*Anopheles stephensi*
*Commensalibacter intestini* A911	PRJNA75109	*Drosophila melanogaster*
*Saccharibacter* sp. AM169	CBLY010000001:9	*Apis mellifera*
*Saccharibacter floricola* DSM 15669^T^	PRJNA181373	Pollen of Japanese flower

### Carbohydrate consumption experiments

To determine the growth rate and glucose consumption rate of strain LMG 28161^T^, one 10-L monoculture fermentation experiment was carried out in basal medium [yeast extract 0.5%, w/v, [24]] supplemented with 1% (w/v) D-glucose. The fermentation was performed in a 15-L BiostatC fermentor (Sartorius AG, Melsungen, Germany) at 28°C, free pH, and 300 rpm for 36 h. Aerobic conditions during the fermentation were ensured by continuously sparging the medium with 5 liters min^-1^ of air. The inoculum for the fermentation experiment was prepared as follows. Strain LMG 28161^T^ was cultivated in 100 mL of LMG medium 404 [5%, w/v, D-glucose and 1%, w/v, yeast extract (Oxoid)] and subsequently propagated twice in 400 mL of basal medium supplemented with 1% (w/v) D-glucose. During the inoculum buildup, the transferred volume was always 5% (v/v). Incubation was done at 28°C for 48 h on a rotary shaker. The inoculum was added to the fermentation vessel aseptically. During the fermentation experiment, the pH was monitored automatically. Samples were withdrawn at regular time intervals for offline analysis.

To verify metabolic pathways, the oxidation of the carbohydrates sucrose, D-glucose, D-fructose, and D-mannitol was verified under aerobic and micro-aerobic conditions (80% N_2_, 4% O_2_, 8% H_2_, and 8% CO_2_). The experiments were conducted in triplicate, using the same method as described previously for acid production from different carbon sources [24], in 50-mL glass bottles filled with 20 mL of basal medium supplemented with 1% (w/v) of the carbon source, but without bromocresol purple added to the medium. The bottles were incubated at 28°C for 7 days on a rotary shaker (aerobic conditions), and in a jar for the micro-aerobic conditions experiments. A medium sample was collected before inoculation and after 7 days of incubation with the culture. The samples were centrifuged and the supernatants were stored at -20C until further analyses were carried out.

### Analysis of bacterial growth, carbohydrate consumption and metabolite production

Growth of LMG 28161^T^ during fermentation [expressed in log (CFU/mL)] was quantified through plating of 10-fold serial dilutions of the samples in physiological solution [0.85% (w/v) NaCl] onto LMG medium M404. Determination of glucose consumption rate was calculated based on the time of glucose depletion, by measuring glucose concentration every three hours as described previously [38]. Determination of carbohydrate, acetic acid, D-gluconic acid, and 2-keto-D-gluconic acid concentrations in the samples taken from the 50-mL bottles, was done using a Focus gas chromatograph (Interscience, Breda, The Netherlands) as described previously [38]. Statistical analysis of carbohydrates consumption was carried out using SPSS version 15.0 (SPSS, Inc., Chicago, IL, USA).

## Supporting Information

S1 Table Group of core orthologs only found among *Asaia platycodi* SF2.1, *Commensalibacter intestini* A911, *Saccharibacter* sp. AM169 and *Saccharibacter floricola* DSM 15669^T^ but not in *Bombella intestini* LMG 28161^T^. Abbrevation “com, sfl, sac” and “asp” before “|” represent *Commensakibacter intestini* A911, *Saccharibacter floricola* DSM 15669^T^, *Saccharibacter* sp. AM169 and *Asaia platycodi* SF2.1, respectively. The number after “|” refers to sequence locus in each of the genome.
(XLSX)

S2 Table Unique genes found in *Bombella intestini* LMG 28161^T^. (XLSX)

## References

[1] Cleenwerck I, González A, Camu N, Engelbeen K, De Vos P, De Vuyst L. *Acetobacter fabarum* sp. nov., an acetic acid bacterium from a Ghanaian cocoa bean heap fermentation. Int J Syst Evol Microbiol. 2008; 58:2180–2185. 10.1099/ijs.0.65778-0 18768626

[2] Illeghems K, De Vuyst L, Weckx S. Complete genome sequence and comparative analysis of *Acetobacter pasteurianus* 386B, a strain well-adapted to the cocoa bean fermentation ecosystem. BMC Genomics. 2013; 14:526 10.1186/1471-2164-14-526 23902333 PMC3751514

[3] Gullo M, Giudici P. Acetic acid bacteria in traditional balsamic vinegar: phenotypic traits relevant for starter cultures selection. Int J Food Microbiol. 2008; 125:46–53. 10.1016/j.ijfoodmicro.2007.11.076 18177968

[4] Raspor P, Goranovič D. Biotechnological applications of acetic acid bacteria. Crit Rev Biotechnol. 2008; 28:101–124. 10.1080/07388550802046749 18568850

[5] Fuentes-Ramirez LE, Bustillos-Cristales R, Tapia-Hernandez A, Jimenez-Salgado T, Wang ET, Martinez-Romero E, et al Novel nitrogen-fixing acetic acid bacteria, *Gluconacetobacter johannae* sp. nov. and *Gluconacetobacter azotocaptans* sp. nov, associated with coffee plants. Int J Syst Evol Microbiol. 2001; 51:1305–1314. 10.1099/00207713-51-4-1305 11491326

[6] Crotti E, Rizzi A, Chouaia B, Ricci I, Favia G, Alma A, et al Acetic acid bacteria, newly emerging symbionts of insects. Appl Environ Microbiol. 2010; 76:6963–6970. 10.1128/AEM.01336-10 20851977 PMC2976266

[7] Wieme AD, Spitaels F, Aerts M, De Bruyne K, Van Landschoot A, Vandamme P. Identification of beer-spoilage bacteria using matrix-assisted laser desorption/ionization time-of-flight mass spectrometry. Int J Food Microbiol. 2014; 185:41–50. 10.1016/j.ijfoodmicro.2014.05.003 24929682

[8] Crotti E, Damiani C, Pajoro M, Gonella E, Rizzi A, Ricci I, et al Asaia, a versatile acetic acid bacterial symbiont, capable of cross-colonizing insects of phylogenetically distant genera and orders. Environ Microbiol. 2009; 11:3252–3264. 10.1111/j.1462-2920.2009.02048.x 19735280

[9] Cariveau DP, Powell JE, Koch H, Winfree R, Moran NA. Variation in gut microbial communities and its association with pathogen infection in wild bumble bees (*Bombus*). ISME J. 2014; 1–11. 10.1038/ismej.2014.68 PMC4260702 24763369

[10] Mohr KI, Tebbe CC. Diversity and phylotype consistency of bacteria in the guts of three bee species (*Apoidea*) at an oilseed rape field. Environ Microbiol. 2006; 8:258–272. 10.1111/j.1462-2920.2005.00893.x 16423014

[11] Martinson VG, Danforth BN, Minckley RL, Rueppell O, Tingek S, Moran NA. A simple and distinctive microbiota associated with honey bees and bumble bees. Mol Ecol. 2011; 20:619–628. 10.1111/j.1365-294X.2010.04959.x 21175905

[12] Cox-Foster DL, Conlan S, Holmes EC, Palacios G, Evans JD, Moran NA, et al A metagenomic survey of microbes in honey bee colony collapse disorder. Science 2007; 318:283–287. 10.1126/science.1146498 17823314

[13] Corby-Harris V, Snyder LA, Schwan MR, Maes P, McFrederick QS, Anderson KE. Origin and effect of Alpha 2.2 *Acetobacteraceae* in honey bee larvae and description of *Parasaccharibacter apium* gen. nov., sp. nov. Appl. Environ. Microbiol. 2014; 80:7460–7472. 10.1128/AEM.02043-14 25239902 PMC4249245

[14] Li L, Praet J, Borremans W, Nunes OC, Manaia CM, Cleenwerck I, et al *Bombella intestini* gen. nov., sp. nov., an acetic acid bacterium isolated from bumble bee crop. Int J Syst Evol Microbiol. 2015; 65:267–273. 10.1099/ijs.0.068049-0 25336723

[15] Stoop J, Williamson J, Masonpharr D. Mannitol metabolism in plants: a method for coping with stress. Trends Plant Sci. 1996; 1:139–144.

[16] Mesbah M, Premachandran U, Whitman WB. Precise measurement of the G+C content of deoxyribonucleic acid by high-performance liquid chromatography. Int J Syst Evol Microbiol. 1989; 39:159–167.

[17] Chouaia B, Gaiarsa S, Crotti E, Comandatore F, Degli Esposti M, Ricci I, et al Acetic acid bacteria genomes reveal functional traits for adaptation to life in insect guts. Genome Biol Evol. 2014; 6:912–920. 10.1093/gbe/evu062 24682158 PMC4007555

[18] Azuma Y, Hosoyama A, Matsutani M, Furuya N, Horikawa H, Harada T, et al Whole-genome analyses reveal genetic instability of *Acetobacter pasteurianus*. Nucleic Acids Res. 2009; 37:5768–5783. 10.1093/nar/gkp612 19638423 PMC2761278

[19] Prust C, Hoffmeister M, Liesegang H, Wiezer A, Fricke WF, Ehrenreich A, et al Complete genome sequence of the acetic acid bacterium *Gluconobacter oxydans*. Nat Biotechnol. 2005; 23:195–200. 10.1038/nbt1062 15665824

[20] Bertalan M, Albano R, de Pádua V, Rouws L, Rojas C, Hemerly A, et al Complete genome sequence of the sugarcane nitrogen-fixing endophyte *Gluconacetobacter diazotrophicus* Pal5. BMC Genomics. 2009; 10:450 10.1186/1471-2164-10-450 19775431 PMC2765452

[21] Kwong WK, Engel P, Koch H, Moran NA. Genomics and host specialization of honey bee and bumble bee gut symbionts. Proc Natl Acad Sci USA. 2014; 111:11509–11514. 10.1073/pnas.1405838111 25053814 PMC4128107

[22] Nilsson AI, Koskiniemi S, Eriksson S, Kugelberg E, Hinton J, Andersson DI. Bacterial genome size reduction by experimental evolution. Proc Natl Acad Sci USA. 2005; 102:12112–12116. 10.1073/pnas.0503654102 16099836 PMC1189319

[23] Bossi RT, Negri A, Tedeschi G, Mattevi A. Structure of FAD-bound L-aspartate oxidase: insight into substrate specificity and catalysis. Biochemistry-US. 2002; 41:3018–3024. 10.1021/bi015939r 11863440

[24] Gosselé F, Swings J, Kersters K, Ley JD. Numerical analysis of phenotypic features and protein gel electropherograms of *Gluconobacter* Asai 1935 emend. mut. char. Asai, Iizuka, and Komagata 1964. Int J Syst Bacteriol. 1983; 33:65–81.

[25] Hölscher T, Görisch H. Knockout and overexpression of pyrroloquinoline quinone biosynthetic genes in *Gluconobacter oxydans* 621H. J Bacteriol. 2006; 188:7668–7676. 10.1128/JB.01009-06 16936032 PMC1636293

[26] Matsutani M, Fukushima K, Kayama C, Arimitsu M, Hirakawa H, Toyama H, et al Replacement of a terminal cytochrome c oxidase by ubiquinol oxidase during the evolution of acetic acid bacteria. Biochim Biophys Acta. 2014; 1837:1810–1820. 10.1016/j.bbabio.2014.05.355 24862920

[27] Low HH, Gubellini F, Rivera-Calzada A, Braun N, Connery S, Dujeancourt A, et al Structure of a type IV secretion system. Nature. 2014; 508:550–553. 10.1038/nature13081 24670658 PMC3998870

[28] Backert S, Tegtmeyer N, Fischer W. Composition, structure and function of the *Helicobacter pylori cag* pathogenicity island encoded type IV secretion system. Future Microbiol. 2015; 10:955–965. 10.2217/fmb.15.32 26059619 PMC4493163

[29] Lohaus G, Schwerdtfeger M. Comparison of sugars, iridoid glycosides and amino acids in nectar and phloem sap of *Maurandya barclayana*, *Lophospermum erubescens*, and *Brassica napus*. PLoS ONE. 2014; 9:e87689 10.1371/journal.pone.0087689 PMC3906183 24489951

[30] Cleenwerck I, De Vos P. Polyphasic taxonomy of acetic acid bacteria: an overview of the currently applied methodology. Int J Food Microbiol. 2008; 125:2–14. 10.1016/j.ijfoodmicro.2007.04.017 18237808

[31] Spitaels F, Wieme A, Balzarini T, Cleenwerck I, Van Landschoot A, De Vuyst L, et al *Gluconobacter cerevisiae* sp. nov., isolated from the brewery environment. Int J Syst Evol Microbiol. 2014; 64:1134–1141. 10.1099/ijs.0.059311-0 24368694

[32] Cleenwerck I, Vandemeulebroecke K, Janssens D, Swings J. Re-examination of the genus *Acetobacter*, with descriptions of *Acetobacter cerevisiae* sp. nov. and *Acetobacter malorum* sp. nov. Int J Syst Evol Microbiol. 2002; 52:1551–1558. 10.1099/00207713-52-5-1551 12361257

[33] Meyer F. GenDB—an open source genome annotation system for prokaryote genomes. Nucleic Acids Res. 2003; 31:2187–2195. 12682369 10.1093/nar/gkg312 PMC153740

[34] Aziz RK, Bartels D, Best AA, DeJongh M, Disz T, Edwards RA, et al The RAST Server: rapid annotations using subsystems technology. BMC Genomics. 2008; 9:75 10.1186/1471-2164-9-75 18261238 PMC2265698

[35] Kanehisa M, Goto S. KEGG: Kyoto encyclopedia of genes and genomes. Nucleic Acids Res. 2000; 28:27–30. 10592173 10.1093/nar/28.1.27 PMC102409

[36] Grissa I, Vergnaud G, Pourcel C. CRISPRFinder: a web tool to identify clustered regularly interspaced short palindromic repeats. Nucleic Acids Res. 2007; 35(Web Server): W52–W57. 10.1093/nar/gkm360 17537822 PMC1933234

[37] Li L, Stoeckert CJ Jr, Roos DS. OrthoMCL: identification of ortholog groups for eukaryotic genomes. Genome Res. 2003, 13:2178–2189. 10.1101/gr.1224503 12952885 PMC403725

[38] Moens F, Lefeber T, De Vuyst L. Oxidation of metabolites highlights the microbial interactions and role of *Acetobacter pasteurianus* during cocoa bean fermentation. Appl Environ Microbiol. 2014; 80:1848–1857. 10.1128/AEM.03344-13 24413595 PMC3957632

